# Post-Infection Immunodeficiency Virus Control by Neutralizing Antibodies

**DOI:** 10.1371/journal.pone.0000540

**Published:** 2007-06-20

**Authors:** Hiroyuki Yamamoto, Miki Kawada, Akiko Takeda, Hiroko Igarashi, Tetsuro Matano

**Affiliations:** 1 International Research Center for Infectious Diseases, The Institute of Medical Science, The University of Tokyo, Tokyo, Japan; 2 Graduate School of Medicine, The University of Tokyo, Tokyo, Japan; 3 AIDS Research Center, National Institute of Infectious Diseases, Tokyo, Japan; 4 Tsukuba Primate Research Center, National Institute of Biomedical Innovation, Ibaraki, Japan; University of California, San Francisco, United States of America

## Abstract

**Background:**

Unlike most acute viral infections controlled with the appearance of virus-specific neutralizing antibodies (NAbs), primary HIV infections are not met with such potent and early antibody responses. This brings into question if or how the presence of potent antibodies can contribute to primary HIV control, but protective efficacies of antiviral antibodies in primary HIV infections have remained elusive; and, it has been speculated that even NAb induction could have only a limited suppressive effect on primary HIV replication once infection is established. Here, in an attempt to answer this question, we examined the effect of passive NAb immunization post-infection on primary viral replication in a macaque AIDS model.

**Methods and Findings:**

The inoculums for passive immunization with simian immunodeficiency virus mac239 (SIVmac239)-specific neutralizing activity were prepared by purifying polyclonal immunoglobulin G from pooled plasma of six SIVmac239-infected rhesus macaques with NAb induction in the chronic phase. Passive immunization of rhesus macaques with the NAbs at day 7 after SIVmac239 challenge resulted in significant reduction of set-point plasma viral loads and preservation of central memory CD4 T lymphocyte counts, despite the limited detection period of the administered NAb responses. Peripheral lymph node dendritic cell (DC)-associated viral RNA loads showed a remarkable peak with the NAb administration, and DCs stimulated in vitro with NAb-preincubated SIV activated virus-specific CD4 T lymphocytes in an Fc-dependent manner, implying antibody-mediated virion uptake by DCs and enhanced T cell priming.

**Conclusions:**

Our results present evidence indicating that potent antibody induction post-infection can result in primary immunodeficiency virus control and suggest direct and indirect contribution of its absence to initial control failure in HIV infections. Although difficulty in achieving requisite neutralizing titers for sterile HIV protection by prophylactic vaccination has been suggested, this study points out a possibility of non-sterile HIV control by prophylactic vaccine-induced, sub-sterile titers of NAbs post-infection, providing a rationale of vaccine-based NAb induction for primary HIV control.

## Introduction

In the natural courses of HIV infections, the host immune responses fail to contain the virus replication and allow persistent plasma viremia. While virus-specific cytotoxic T lymphocyte (CTL) responses exert strong suppressive pressure on primary HIV replication [1]–[7], the contribution of virus-specific antibodies in clearance of primary HIV infection has remained unclear [8].

Neutralizing antibodies (NAbs) play a central role in control of most viral infections, but in HIV infections, NAb induction is not efficient in the early phase due to its unusual neutralization-resistant nature, such as the sophisticated masking of neutralizing epitopes in HIV envelope [8]–[11], and protective efficacies of post-infection NAbs in vivo have remained elusive. While evidence of virus escape implies NAb selective pressure to a certain extent [10], [12]–[13], it has been speculated that post-infection NAbs could exert only a limited suppressive effect on primary HIV replication [14]–[16].

Post-infection passive NAb immunization studies in macaque AIDS models would contribute to elucidation of its protective role, in complementation with studies determining the requisites for sterile protection by pre-challenge administered NAb titers [14], [16]–[21]. A model of CCR5-tropic simian immunodeficiency virus (SIV) infection that induces acute loss of memory CD4^+^ T cells like HIV infections in humans [22]–[25] would be adequate for assessment of post-infection NAb efficacies in primary immunodeficiency virus infection.

In the present study, we examined the effect of passive NAb immunization at day 7 post-challenge on primary viral replication in a macaque AIDS model of CCR5-tropic SIVmac239 infection. Remarkably, our analysis revealed control of primary SIVmac239 replication by the passive NAb immunization post-infection.

## Methods

### Animal experiments

Burmese rhesus macaques (*Macaca Mulatta*) were maintained in accordance with the Guideline for Laboratory Animals of National Institute of Infectious Diseases and National Institute of Biomedical Innovation. Major histocompatibility complex class I (MHC-I) haplotypes were determined by reference strand-mediated conformation analysis as described previously [6], [26]. Blood collection, vaccination, virus challenge, passive immunization, and lymph node biopsy were performed under ketamine anesthesia. For vaccination, animals intramuscularly received a priming with 5 mg of CMV-SHIVdEN DNA encoding SIVmac239 Gag, Pol, Vif, and Vpx, SIVmac239-HIV-1_DH12_ chimeric Vpr, and HIV-1_DH12_ Tat and Rev, followed by an intranasal booster six weeks later with 1×10^8^ CIU (cell infectious units) of replication-competent Sendai virus expressing Gag (SeV-Gag) in macaque V5 or 6×10^9^ CIU of F-deleted replication-defective SeV-Gag in other vaccinees as described previously [6]. Animals were challenged intravenously with 1,000 TCID_50_ (50 percent tissue culture infective dose) of SIVmac239, three months after booster in case of vaccinees. For passive immunization, animals were intravenously administered with 300 mg of anti-SIV immunoglobulin G (IgG) or control IgG at day 7 post-challenge.

### Antibody preparation

Pools of plasma showing SIVmac239-specific NAb titers of 1∶4 to 1∶64 were obtained from six SIVmac239-infected rhesus macaques with NAb induction in the chronic phase for preparing the IgG inoculums for passive NAb immunization. IgG was purified from the plasma after heat-inactivation and filtration by Protein G Sepharose 4 Fast Flow (Amersham) and concentrated by Amicon Ultra 4, MW50000 (Millipore) to 30 mg/ml. This IgG solution had SIVmac239-specific NAb titer of 1∶16; i.e., 5 µl of 16-fold-diluted antibodies killed 5 µl of 10 TCID_50_ SIVmac239 on MT-4 cells. Control IgG was prepared from non-infected rhesus macaques. Neutralizing F(ab')2 was obtained by pepsin digestion with Immunopure F(ab')2 purification kit (Pierce).

### Quantitation of plasma viral loads

Plasma RNA was extracted using High Pure Viral RNA kit (Roche Diagnostics). Serial five-fold dilutions of RNA samples were amplified in quadruplicate by reverse transcription and nested PCR using SIVmac239 *gag*-specific primers to determine the end point. Plasma SIV RNA levels were calculated according to the Reed-Muench method as described previously [6]. The lower limit of detection is approximately 4×10^2^ copies/ml.

### Measurement of virus-specific neutralizing titers

Serial two-fold dilutions of heat-inactivated plasma or purified antibodies were prepared in duplicate and mixed with 10 TCID_50_ of SIVmac239. In each mixture, 5 µl of diluted sample was incubated with 5 µl of virus. After 45-min incubation at room temperature, each 10-µl mixture was added into 5×10^4^ MT-4 cells/well in 96-well plates. Day 12 culture supernatants were harvested and progeny virus production was examined by ELISA for detection of SIV p27 core antigen (Beckman-Coulter) to determine 100% neutralizing endpoint. The lower limit of titration is 1∶2.

### Measurement of virus-specific T-cell responses

Virus-specific T-cell levels were measured by flow-cytometric analysis of gamma interferon (IFN-γ) induction as described previously [6]. Peripheral blood mononuclear cells (PBMCs) were cocultured with autologous herpesvirus papio-immortalized B lymphoblastoid cell lines infected with a vesicular stomatitis virus G (VSV-G)-pseudotyped SIVGP1 for SIV-specific stimulation. The pseudotyped virus was obtained by cotransfection of COS-1 cells with a VSV-G-expression plasmid and the SIVGP1 DNA, an env- and nef-deleted simian-human immunodeficiency virus (SHIV) molecular clone DNA. Intracellular IFN-γ staining was performed using CytofixCytoperm kit (Becton Dickinson). Fluorescein isothiocianate-conjugated anti-human CD4, Peridinin chlorophyll protein-conjugated anti-human CD8, allophycocyanin-conjugated anti-human CD3, and phycoerythrin-conjugated anti-human IFN-γ antibodies (Becton Dickinson) were used. Specific T-cell levels were calculated by subtracting non-specific IFN-γ^+^ T-cell frequencies from those after SIV-specific stimulation. Specific T-cell levels less than 100 cells per million PBMC are considered negative.

### Quantitation of cell-associated viral loads

Right and left inguinal lymph nodes and right and left axillary lymph nodes were obtained from macaques by biopsy at days 7, 8, 10, and 14 post-challenge, respectively. For measurement of dendritic cell (DC)-associated viral loads, CD1c^+^ DCs were positively selected to over 99% purity using a macaque CD1c^+^ DC magnetic sorting system (Miltenyi Biotech) from CD20^−^ lymphocytes negatively-selected from lymph nodes. CD1c^−^CD20^−^ cells were used for measurement of non-DC-associated viral loads. Cell-associated viral RNA was extracted using RNeasy kit (Qiagen) and quantitated by LightCycler real-time PCR system (Roche Diagnostics) using SIV *gag*-specific primers and probes. The lower limit of detection is approximately 1,000 copies/10^6^ cells.

### Antigen presentation assay in vitro

PBMCs obtained in the chronic phase from SIVmac239-controllers were attached to culture plates for 4 h, and adhesive cells were cultured in the presence of 50 ng/ml GM-CSF (R&D Systems) and 5 ng/ml IL-4 (R&D Systems) for 5 days to obtain CD1c^+^CD83^+^CD86^+^HLA-ABC^+^HLA-DR^+^ immature DCs [27]. Alternatively, CD1c^+^ DCs were positively selected from CD20-depleted PBMCs as described above. For antigen presentation assay, 1×10^5^ of the in vitro-generated DCs (Exp. 1, 2, and 3) or the positively-selected CD1c^+^ DCs (Exp. 4) were pulsed for 17 h with 2,000 TCID_50_ of SIVmac239 (corresponding to 2×10^6^ SIV RNA copies and 3 ng of SIV p27) alone or preincubated for 45 min with 1.5 mg of either control IgG, neutralizing IgG, or neutralizing F(ab')2. Autologous PBMCs were cocultured with these pulsed DCs and then subjected to measurement of specific IFN-γ induction.

### Statistical analysis

Statistical analysis was performed by Prism software version 4.03 (GraphPad Software, Inc.). Set point plasma viral loads and peripheral CD95^+^CD28^+^ central memory CD4^+^ T-cell counts around 3 months after challenge of the naive controls (n = 7) and NAb-immunized macaques (n = 4) were log-transformed for improvement of normality and compared by two-tailed unpaired t test with significance levels set at *p*<0.05. Then their geometric means with 95% confidence interval were calculated. Due to the limited number of samples for each group providing difficulty for their normality testing, the two groups were additionally compared by nonparametric Mann-Whitney U test for confirmation of results. No significant difference in CD95^+^CD28^+^ central memory CD4^+^ T-cell counts just before challenge was observed between the two groups (*p* = 0.68 by unpaired two-tailed t test with Welch's correction and *p* = 0.31 by Mann-Whitney U test) (data not shown).

## Results

### SIV control by post-infection passive NAb immunization

**Figure 1:**
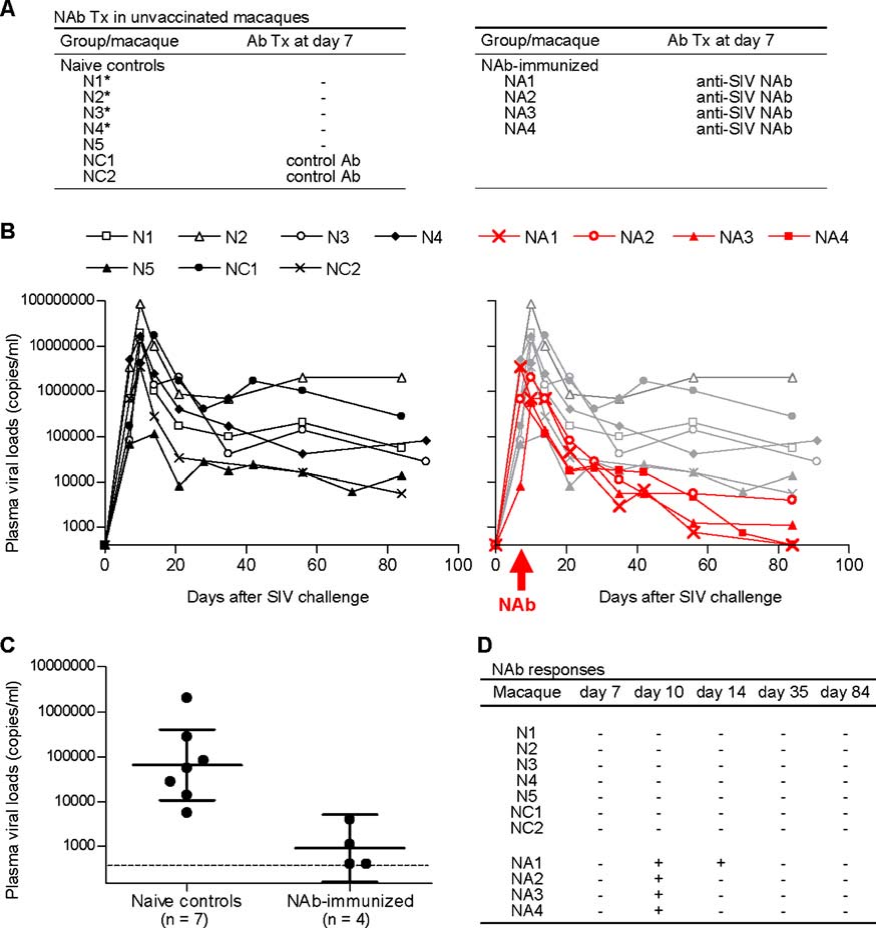
Effect of post-challenge passive NAb immunization on primary SIV infection. (A) List of naive controls and NAb-immunized macaques. Experiments using macaques indicated by asterisk have previously been performed [6]. (B) Plasma viral loads after SIVmac239 challenge (SIV RNA copies/ml). Left panel, naive controls; right panel, NAb-immunized macaques shown by red lines and naive controls by gray lines for comparison. (C) Statistical analysis of plasma viral loads around 3 months post-challenge between naive controls (n = 7) and NAb-immunized macaques (n = 4). The geometric mean (indicated by the longer bar) of viral loads in naive controls is 6.5×10^4^ copies/ml, and its 95% confidence interval (indicated by the shorter bars) is 1.1×10^4^−4.0×10^5^ copies/ml. The geometric mean in NAb-immunized macaques is 9.1×10^2^ copies/ml, and its 95% confidence interval is 1.6×10^2^−5.1×10^3^ copies/ml. The difference between the two groups was statistically significant by unpaired two-tailed t test (*p* = 0.0033) and by non-parametric Mann-Whitney U test (*p* = 0.0061). Viral loads of macaques NA1 and NA4 were calculated as the lower limit of detection shown as the dashed line (400 copies/ml). (D) Plasma NAb responses after challenge. (+), positive; (−), negative. All detected titers were no more than 1:2.

While most SIVmac239-infected naive macaques usually fail to elicit NAb responses during the early phase of infection, some acquire detectable levels of NAbs against the challenge strain in the late phase. IgG purified from plasma pools of such SIVmac239-infected macaques with NAb induction, showing in vitro SIVmac239-specific neutralizing activity of 1∶16, was used for passive immunization as polyclonal anti-SIV NAbs. In the first part of this study, naive Burmese rhesus macaques were challenged intravenously with SIVmac239 followed by passive immunization with 10 ml of the polyclonal NAbs (300 mg IgG) at day 7 post-challenge (Figure 1A). Seven naive control macaques challenged with SIVmac239, including two infused with non-SIV-specific control antibodies, all failed to contain viral replication with persistent viremia (Figure 1B). These macaques showed peak plasma viral loads between days 7 and 14 post-challenge and most had set-point viral loads exceeding 1×10^4^ SIV RNA copies/ml plasma. In contrast, four rhesus macaques passively immunized at day 7 with polyclonal NAbs showed significantly lower plasma viral RNA loads (*p* = 0.0033 by unpaired t test and *p* = 0.0061 by Mann-Whitney U test) compared with naive controls around 3 months post-challenge (Figures 1B&1C). Two of the NAb-immunized macaques, NA1 and NA4, controlled SIV replication with undetectable set-point plasma viremia. Thus, post-infection passive immunization of macaques with polyclonal NAbs had a significant suppressive effect on set-point viral replication.

### Immune parameters in NAb-immunized macaques

Plasma NAb responses in the NAb-immunized macaques were detected marginally at day 10 post-infection but became undetectable within one week after the passive NAb immunization (Figure 1D), implying that the NAbs were rapidly exhausted for virus clearance. None elicited detectable *de novo* NAb responses past then. In the naive controls, no SIVmac239-specific NAbs were detected throughout the course. This discrepancy between the transient NAb detection and the persistent viremia control in the NAb-immunized macaques differed from previously-reported, dose-dependent establishment of sterile protection from CXCR4-tropic SHIV infection by pre-challenge passive NAb immunization [18]–[21].

**Figure 2:**
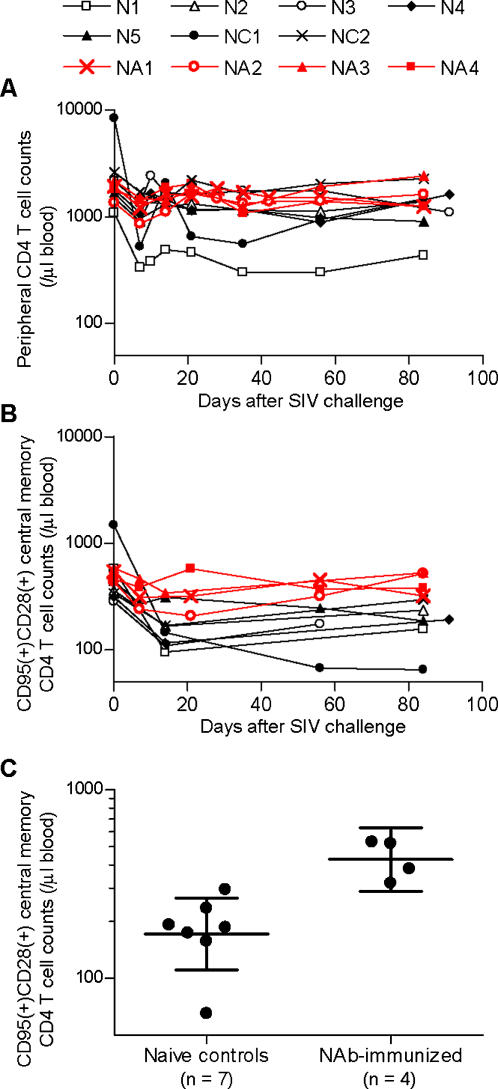
Central memory CD4^+^ T-cell counts in naive controls and NAb-immunized macaques. (A) Peripheral CD4^+^ T-cell counts (cells/µl). (B) Peripheral CD95^+^CD28^+^ central memory CD4^+^ T-cell counts (cells/µl) [28]. (C) Statistical comparison of CD28^+^CD95^+^ central memory CD4^+^ T-cell counts around 3 months post-challenge. The geometric mean (indicated by the longer bar) of central memory CD4^+^ T-cell counts in naive controls is 1.7×10^2^ counts/µl, and its 95% confidence interval (indicated by the shorter bars) is 1.1×10^2^−2.7×10^2^ counts/µl. The geometric mean in NAb-immunized macaques is 4.3×10^2^ counts/µl, and its 95% confidence interval is 2.9×10^2^−6.3×10^2^ counts/µl. The difference between the two groups was statistically significant by unpaired two-tailed t test (*p* = 0.0066) and by non-parametric Mann-Whitney U test (*p* = 0.0061).

**Figure 3:**
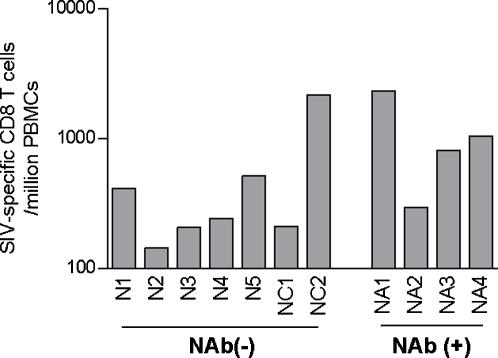
SIV-specific CD8^+^ T-cell frequencies at week 8 post-challenge in naive controls and NAb-immunized macaques.

Difference in total CD4^+^ T-cell counts was not found throughout the course between the two groups (Figure 2A). Reductions in peripheral CD95^+^ CD28^+^ central memory CD4^+^ T-cell counts [28]–[29] were observed in the naive controls after SIV challenge (Figure 2B). The NAb-immunized macaques, however, showed significantly higher central memory CD4^+^ T-cell counts around 3 months post-challenge than those in the naive controls (*p* = 0.0066 by unpaired t test and *p* = 0.0061 by Mann-Whitney U test) (Figures 2B&2C), suggesting amelioration of central memory CD4^+^ T-cell loss in the early phase of SIV infection by transient NAb responses around week 1 post-challenge. All of these NAb-immunized macaques showed efficient virus-specific CD8^+^ T-cell induction at week 8 (Figure 3), although difference in the levels between the two groups was not significant, implying its possible enrollment in the observed viral control.

### Post-infection passive NAb immunization in vaccinees

**Figure 4:**
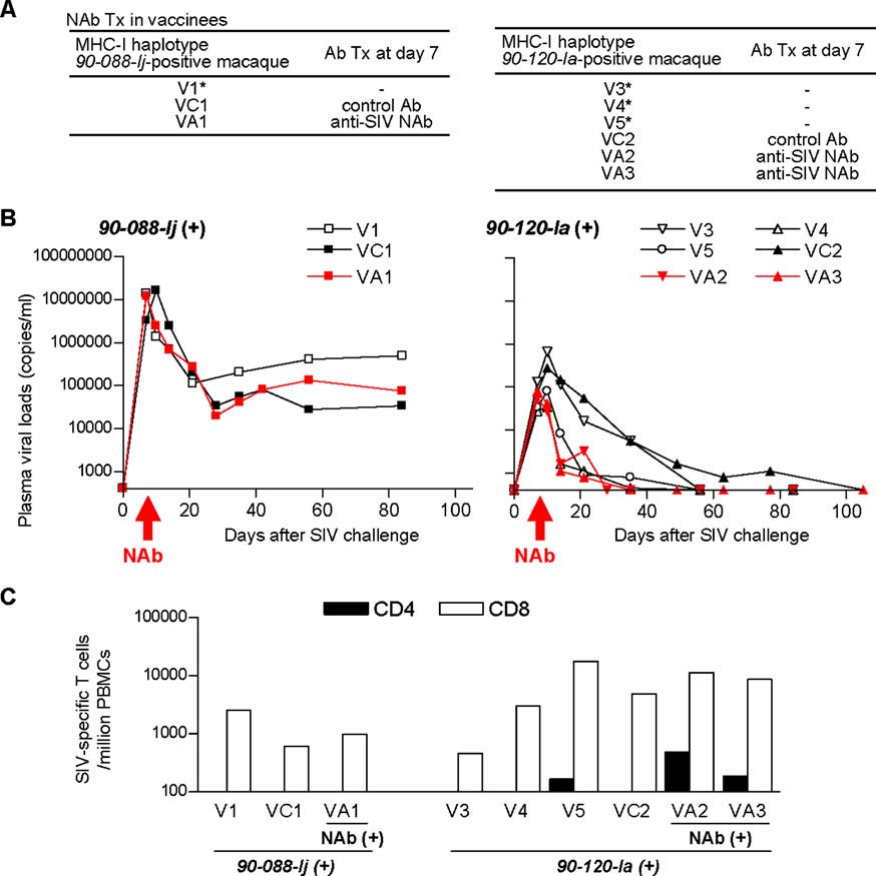
Effect of post-challenge passive NAb immunization in vaccinees. (A) List of vaccinees with or without passive immunization. (B) Plasma viral loads after challenge (SIV RNA copies/ml). Left panel, MHC-I haplotype *90-088-Ij*-positive macaques; right panel, *90-120-Ia*-positive macaques. Red lines represent NAb-immunized vaccinees. (C) SIV-specific CD4^+^ T-cell and CD8^+^ T-cell frequencies at week 2 post-challenge.

Our previous trial of a DNA-prime/SeV-Gag vector-boost vaccine in Burmese rhesus macaques has shown vaccine-based, NAb-independent control of SIVmac239 replication, suggesting association of MHC-I haplotype with this control [6], [30]. We then examined possible synergy of post-challenge passive NAb immunization with the prophylactic CTL-based vaccination in suppression of SIV replication in two groups of macaques possessing MHC-I haplotype *90-088-Ij* and *90-120-Ia*, respectively (Figure 4A). In the former group of macaques possessing *90-088-Ij*, vaccinees failed to control SIV replication even after passive NAb immunization (Figure 4B). In the latter group of macaques possessing *90-120-Ia*, all 4 vaccinees without NAb immunization controlled SIVmac239 replication and had undetectable plasma viral loads after week 8 post-challenge (Figure 4B). All of them rapidly selected for a mutation escaping from Gag_206-216_ epitope-specific CTL by week 5, suggesting a strong selective pressure on the virus by this CTL [6]. As for the two vaccinees VA2 and VA3 infused with NAbs, plasma viremia became undetectable by week 5 and rapid selection of CTL escape mutation was not observed (data not shown). SIV-specific CD8^+^ T-cell frequencies at week 2 in the NAb-immunized vaccinees VA2 and VA3 were comparable with the vaccinees without NAb immunization, while SIV-specific CD4^+^ T-cell induction at week 2 was observed in just one (V5) of the four vaccinees without NAb but in both of the NAb-immunized vaccinees (Figure 4C). These results suggest, even in the NAb-immunized vaccinees, a dominant effect of vaccine-induced cellular immune responses on control of SIV replication, although implying a possibility of NAb-mediated augmentation of CTL vaccine-based viral control.

### Antibody-mediated virion uptake by DCs and T cell priming

**Figure 5:**
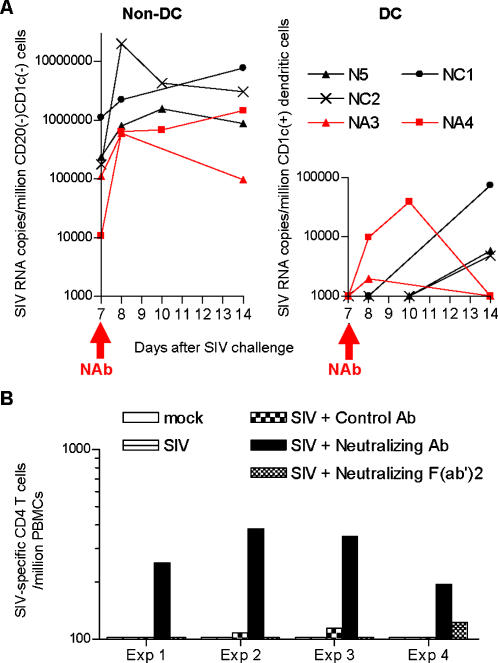
Antibody-mediated SIV uptake by DCs and T cell priming. (A) Peripheral lymph node-derived non-DC (CD1c^−^CD20^−^ lymphocytes)-associated (left panel) and CD1c^+^CD20^−^ DC-associated viral loads (right panel). (B) In vitro antigen presentation assay. Either in vitro-generated DCs (Exp. 1, Exp. 2, and Exp. 3) or positively-selected CD1c^+^ DCs (Exp. 4) prepared from PBMCs were pulsed with SIV alone (SIV), SIV preincubated with control antibodies (SIV+Control Ab), SIV preincubated with NAbs (SIV+Neutralizing Ab), or SIV preincubated with Fc-depleted NAbs (SIV+Neutralizing F[ab']2). Autologous PBMCs were cocultured with these pulsed DCs and then subjected to measurement of specific IFN-γ induction.

In order to assess the possibility of altered virus distribution by NAbs, CD1c^+^ DCs were isolated from peripheral lymph nodes of unvaccinated, SIVmac239-challenged macaques before and after passive NAb immunization, and DC-associated SIV RNA levels were quantified at the initial stage of infection. In three naive control macaques, accumulation of viral RNA to CD1c^+^ DCs was undetectable at days 7, 8, and 10 post-challenge but became detectable at day 14 (Figure 5A). This elevation of DC-associated viral loads following peak viremia was consistent with previous immunohistochemistry reports on SIV and HIV-2 challenge experiments [31]–[32]. In marked contrast, both of macaques NA3 and NA4 immunized with NAbs at day 7 post-challenge showed immediate accumulation of viral RNA in CD1c^+^ DCs at day 8 (one day after NAb immunization), suggesting antibody-mediated virion accumulation to DCs in vivo. Cell-associated viral loads in CD1c^−^CD20^−^ non-DCs were at comparable levels between the two groups, indicating that the rapid increase in DC-associated viral loads after NAb immunization was not due to changes in viral loads in lymph nodes.

Then an in vitro antigen presentation assay was performed to assume whether the early viral RNA accumulation in DCs could represent a correlation to T cell priming. DCs prepared from peripheral blood of macaques that controlled SIVmac239 replication were pulsed with antibody-neutralized SIV, and autologous PBMCs were cocultured with these pulsed DCs for measurement of specific IFN-γ induction. In all four sets of experiments, efficient IFN-γ induction in CD4^+^ T cells was observed after stimulation by DCs pulsed with SIV preincubated with NAb but not by DCs pulsed with SIV alone, SIV preincubated with control antibodies, or SIV preincubated with Fc-depleted neutralizing F(ab')2 (Figure 5B). Efficient IFN-γ induction in CD8^+^ T cells was not observed even after coculture with NAb-preincubated SIV-pulsed DCs except for one (Exp. 4). Overall, augmentation of virus-specific T-cell stimulation was observed by the coexistence of NAbs, suggesting their involvement in antigen presentation.

## Discussion

The present study showed suppression of primary SIV replication by passive NAb immunization post-infection, suggesting a possibility of HIV control by potent antibody induction during the acute phase of infection. It reversely follows that its absence may be involved in an increase in the burden of acute infectious viral loads and abrogation of virus-specific cellular immune responses, leading to initial control failure in HIV infections.

While this study does not exclude possibilities of additional antibody-mediated protective mechanisms such as antibody-dependent cell-mediated cytotoxicity or recently-reported complement virolysis [33], the non-sterile but consistent viral control at the set point by passive NAb immunization despite only transient detection of NAb responses during the acute phase coheres with involvement of cellular immune responses in this control [27], [34]–[36]. Thus, results may provide additional interpretations to previous NAb passive immunization studies [14], [16]–[21], which have mostly utilized CXCR4-tropic SHIV-challenged macaques and shown sterile protection by high titers of pre-challenge or very early post-challenge NAbs.

A technical confinement of this study is the use of polyclonal antibodies which may include not only NAbs but also non-neutralizing anti-SIV antibodies for passive immunization. However, our finding of primary SIV control by post-infection passive immunization with the anti-SIV inoculums with neutralizing activity presents significant evidence suggesting that potent antibodies post-infection can contribute to control of primary immunodeficiency virus infection. Whether neutralizing activity is required for the enhanced SIV control by passive immunization remains to be assessed in future studies. Our in vitro results suggest a possibility of virus-specific CD4^+^ T-cell activation by NAbs, and neutralizing activity may contribute to protection of these virus-specific CD4^+^ T cells from SIV *trans*-infection via DCs [37]–[38], possibly counteracting the abrogation of the optimal concert of adaptive immunity between CD4^+^ T and CD8^+^ T cells usually observed in the natural course of pathogenic immunodeficiency virus infection [23], [25], [39]. The possibility of failure in antibody-mediated priming of effective cellular immune responses by preexisting vaccine-induced dominant responses may account for lack of viral control in the NAb-immunized vaccinee possessing MHC-I haplotype *90-088-Ij*.

Despite suggested technical difficulties in achieving requisite neutralizing titers for sterile HIV protection by prophylactic vaccination, our results indicate a possibility of non-sterile HIV control by secondary expansion of prophylactic vaccine-induced, sub-sterile titers of NAbs post-infection, providing a rationale of vaccine-based NAb induction for primary HIV control. More understanding of the mechanism may lead to a more certain rationale for careful induction of NAbs and CTLs by vaccination, maybe potentially capable of synergistic HIV-1 control.
